# Individualized blood pressure management and postoperative organ dysfunction in older hip fracture patients: a study protocol for a single-center, randomized, controlled trial

**DOI:** 10.1186/s12877-026-07594-5

**Published:** 2026-05-02

**Authors:** WeiRan Zhang, NingNing Fang, XiuYu Wang, Lian Liu, ShaoZhong Yang

**Affiliations:** 1 Department of Anesthesiology, Qilu Hospital of Shandong University, No. 107, Wenhua West Road, Jinan, Shandong Province 250012 China; 2 Department of Orthopedic, Qilu Hospital of Shandong University, Ji’nan, China

**Keywords:** Older patients, Hip fracture surgery, Intraoperative hypotension, Individualized blood pressure management, Multiple organ system dysfunction

## Abstract

**Background:**

Postoperative organ dysfunction is a leading cause of death and disability following hip fracture surgery in older patients. Intraoperative hypotension is a major modifiable risk factor for this complication, yet the optimal management strategy to prevent it remains controversial. We hypothesize that an individualized blood pressure management strategy is superior to standard management in reducing postoperative organ dysfunction.

**Methods:**

This single-center, randomized, controlled trial will enroll 180 patients aged 65–85 years with hip fractures under general anesthesia. Eligible patients will be randomly allocated in a 1:1 ratio to the individualized management group (targeting systolic blood pressure within ± 10% of baseline) or the standard management group (reactive management, where intervention is initiated only if systolic blood pressure < 90 mmHg or decrease of > 30% from baseline). A universal mean arterial pressure target of ≥ 65 mmHg will be maintained for all patients. The allocated hemodynamic management strategy will be maintained throughout surgery and during the post-anesthesia care unit stay. The primary outcome is a composite of dysfunction in at least one organ system (respiratory, cardiovascular, renal, and neurological) within 7 days after surgery. Secondary outcomes include the components of the primary outcome, intraoperative variables (including hemodynamic management data, fluid balance, blood loss, and serum lactate levels), intensive care unit and hospital stay, and all-cause mortality within 30 days after surgery.

**Discussion:**

This randomized controlled trial aims to determine whether individualized blood pressure management reduces postoperative organ dysfunction more effectively than standard management in older hip fracture surgery patients. If proven effective, this proactive approach may represent a significant advance in clinical practice, moving from reactive hypotension correction to preventive stabilization, potentially reducing major complications, shortening hospital stays, and improving functional recovery. The results will provide important evidence to guide hemodynamic management during general anesthesia in this vulnerable population, contributing to standardized, evidence-based protocols for enhancing perioperative outcomes.

**Trial registration:**

Trial registration: Chinese Clinical Trial Registry, ChiCTR2400093838. Registered on 12 December 2024. This manuscript presents the study protocol; participant recruitment is ongoing and results will be reported upon trial completion.

**Supplementary Information:**

The online version contains supplementary material available at 10.1186/s12877-026-07594-5.

## Background

Hip fracture is a common and serious injury among older patients, associated with a one-year mortality rate as high as 30% [1]. Survivors often face long-term challenges, including multiple systemic complications, disability, increased risk of refracture, and substantial healthcare costs [2].

The anesthetic management of hip fracture surgery commonly involves either neuraxial or general anesthesia. While both are acceptable, general anesthesia is frequently preferred or necessitated in cases of patient agitation, anticoagulation, difficult positioning, or anticipated prolonged and complex surgery [3–5]. However, general anesthetic agents can induce significant arterial and venous vasodilation, reduce systemic vascular resistance, and cause hemodynamic instability [6]. These effects are compounded by the age-related impairment of cardiovascular autoregulation and a high prevalence of comorbidities, which collectively render older hip fracture patients particularly susceptible to intraoperative hypotension (IOH) [7].

IOH is a well-established risk factor for hypoperfusion of vital organs, leading to adverse outcomes such as postoperative delirium [8], acute kidney injury (AKI) [9, 10] and myocardial injury [11], ultimately worsening the prognosis. The prevalence of IOH in this population is high. A large UK study reported that 61.0% of older hip fracture patients experienced a mean arterial pressure (MAP) < 65 mmHg, and 91.3% had a reduction in systolic blood pressure (SBP) > 20% from baseline [12].

Despite its high prevalence and strong association with poor outcomes, there is no consensus on the optimal definition or management strategy for IOH. Current guidelines primarily recommend avoiding excessive hypotension (e.g., a > 30% decrease from baseline or an absolute SBP < 90 mmHg) and maintaining MAP ≥ 65 mmHg [13, 14]. This approach is inherently reactive, as treatment is initiated only after hypotension occurs. Furthermore, the fixed threshold of MAP ≥ 65 mmHg may not be optimal for all patients, as it fails to account for individual variations in baseline blood pressure and autoregulatory thresholds.

In contrast to this reactive approach, an emerging paradigm is individualized or preemptive blood pressure management, which aims to maintain SBP within a narrow range (e.g., ± 10%) of baseline. This strategy has shown promise in reducing postoperative organ dysfunction in patients undergoing major abdominal surgery [15, 16], suggesting that proactive stabilization may be superior to reactive correction. However, high-quality evidence from randomized controlled trials is lacking specifically in older hip fracture patients.

Therefore, we hypothesize that an individualized blood pressure management strategy (targeting SBP within ± 10% of baseline) will reduce the incidence of postoperative organ dysfunction compared to a standard management strategy (preventing SBP < 90 mmHg or a decrease of > 30% from baseline) in older patients undergoing hip fracture repair under general anesthesia.

## Methods

### Study design

**Fig.1:**
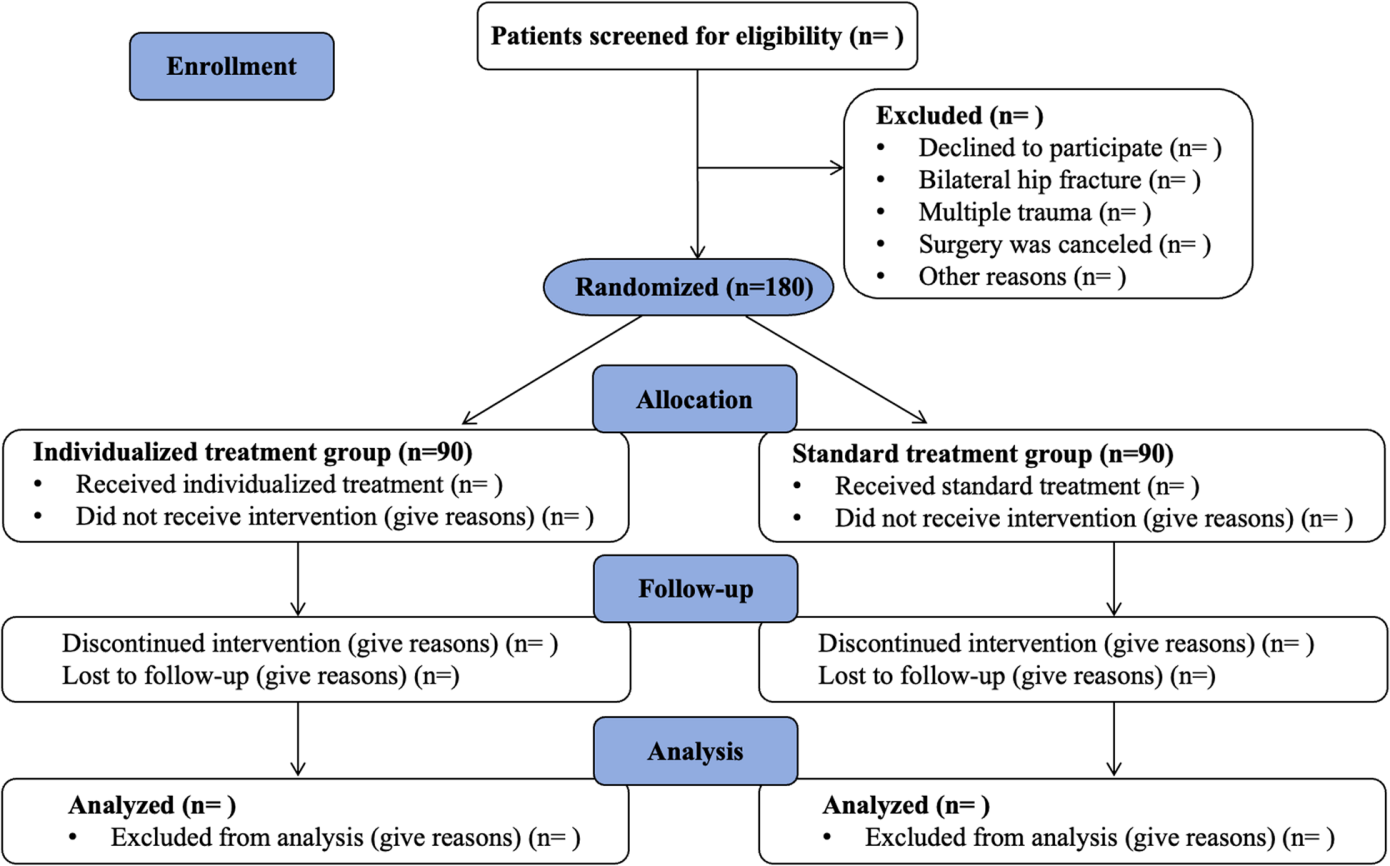
Simplified schematic of the trial design

This randomized controlled trial was approved by the Medical Ethics Committee of Qilu Hospital of Shandong University (No. KYLL-202406–008-2; October 9, 2024) and registered at the Chinese Clinical Trial Registry (ChiCTR2400093838, December 12, 2024). This study has been conducted since December 2024 and will be completed by June 2026 in accordance with the Declaration of Helsinki and the Standard Protocol Items for Randomized Trials (SPIRIT) guidelines. A total of 180 participants will be randomly assigned to either an individualized or a standard treatment group. The research pattern is shown in Fig. 1. The participant timeline is demonstrated in Table 1.

**Table 1:**
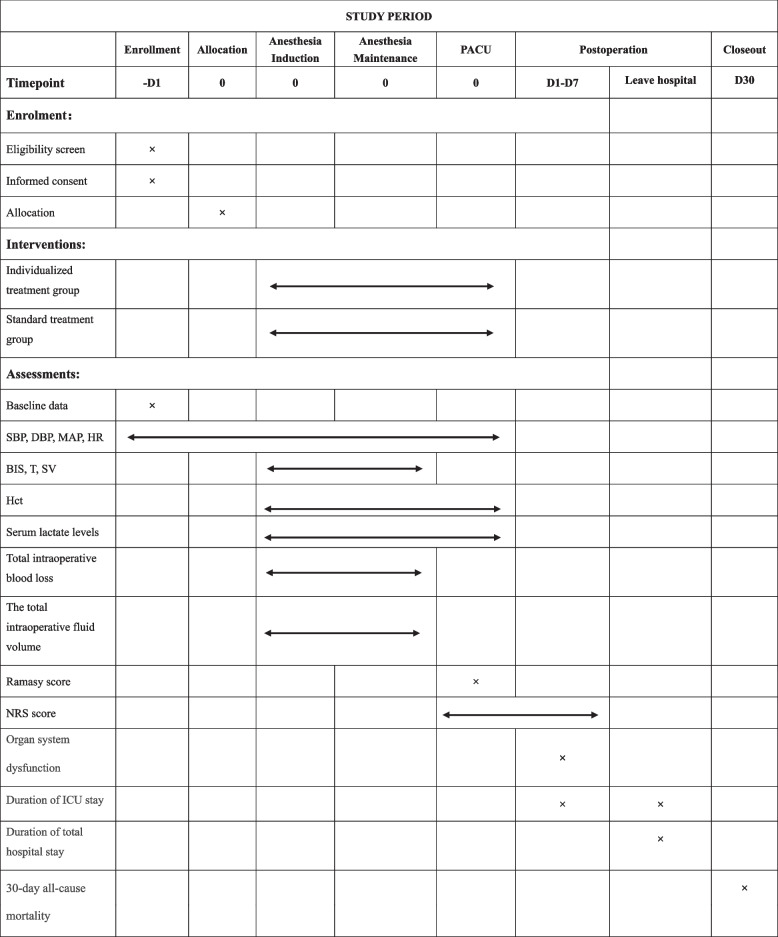
Timeline of the study

*Abbreviations:* *PACU* postanesthesia care unit, *SBP* Systolic blood pressure, *DBP* Diastolic blood pressure; MAP, Mean arterial pressure, *HR* heart rate, *BIS* Bispectral Index, *T* temperature, *SV* Stroke volume, *Hct* hematocrit, *NRS* numerical rating scale, *ICU* Intensive Care Unit

### Trial objectives

The primary objective of this study is to determine whether individualized blood pressure management reduces the incidence of a composite of organ dysfunction within 7 days after surgery compared with standard management in older hip fracture patients.

### Research objects

Older patients (aged 65–85 years) who are scheduled to undergo hip fracture surgery under general anesthesia.

#### Inclusion criteria

1. Age between 65 and 85 years;
2. American Society of Anesthesiologists (ASA) grades I-III;
3. Patients who are planning to undergo surgery for a unilateral hip fracture under general anesthesia;
4. Provision of signed written informed consent from the patient or their legally authorized representative.

#### Exclusion criteria

1. Severe uncontrolled hypertension (SBP ≥ 180 mmHg or diastolic blood pressure [DBP] ≥ 110 mmHg) at rest before surgery;
2. History of acute cardiovascular or cerebrovascular events (e.g., acute myocardial infarction, heart failure, acute cerebral infarction) within 30 days prior to surgery;
3. Previous history of major neurological or psychiatric disorders that would preclude accurate assessment of postoperative delirium or cognitive function, including but not limited to: moderate to severe Alzheimer’s disease, Parkinson's disease, prior stroke with significant residual cognitive or functional deficit, or psychosis;
4. Severe pre-existing organ dysfunction, defined as any of the following:
  - Cardiac: New York Heart Association (NYHA) class IV heart failure or left ventricular ejection fraction (LVEF) < 30%.
  - Respiratory: Home oxygen therapy for chronic obstructive pulmonary disease (COPD) or forced expiratory volume in 1 s (FEV1) < 50% of predicted.
  - Hepatic: Child–Pugh class C liver cirrhosis.
  - Renal: Estimated glomerular filtration rate (eGFR) < 30 mL/min/1.73m^2^ or on chronic renal replacement therapy (dialysis).
5. Multiple fractures or other trauma;
6. Requirement for any vasopressor or inotrope infusion at the time of screening or hospital admission for the hip fracture, regardless of duration or indication;
7. Current participation in another interventional clinical trial;
8. Inability to communicate effectively, or severe hearing/visual impairment preventing reliable outcome assessment.

#### Withdrawal criteria

1. Participant Initiated: Participants may withdraw at any time without penalty. Reasons will be documented if provided.
2. Investigator Initiated: The principal investigator may discontinue a participant for:
  - Major protocol deviation (e.g., unplanned change in anesthesia or surgery).
  - Loss to follow-up (inability to complete the 7-day primary outcome assessment).
  - A serious perioperative complication where continuation is not in the participant's best interest.
  - A Serious Adverse Event (SAE) probably or definitely related to the intervention.
3. Data Handling: The primary analysis will follow the intention-to-treat (ITT) principle. Data collected until withdrawal will be included unless consent for data use is explicitly revoked.

### Randomization and blinding

Eligible participants will be randomly allocated in a 1:1 ratio to either the individualized or standard management group. The randomization sequence will be computer-generated by an independent statistician using block randomization with varying block sizes (of 4 and 6), stratified only by age group (65–74 years vs. 75–85 years) to ensure balance across this key prognostic factor. To guarantee allocation concealment and balance between groups throughout the trial, sequentially numbered, opaque, sealed envelopes will be used. The envelopes will be stored by an independent coordinator and opened by the attending anesthesiologist immediately prior to anesthesia induction. Due to the nature of the intervention, the anesthesiologist performing the intervention cannot be blinded. However, participants, postoperative outcome assessors, and data analysts will remain blinded to group assignment throughout the trial.

## Intervention

### Preoperative interview

Within 24 h prior to surgery, eligible patients will undergo a comprehensive preoperative assessment. This includes evaluation of general status, medical history, physical examination, and laboratory results to determine ASA physical status and Clinical Frailty Scale (CFS) score. The study's purpose, procedures, risks, and benefits will be explained in detail to the patient and their legally authorized representative. Written informed consent for both routine anesthesia and trial participation will be obtained.

The reference blood pressure will be obtained under standardized, pain-stabilized conditions. On the day before surgery, after achieving effective analgesia (Numerical Rating Scale [NRS] score ≤ 3) with Flurbiprofen Cataplasms (initiated upon admission and supplemented with rescue opioids as needed), the patient will rest supine for 5 min. Three consecutive SBP measurements will then be taken at 2-min intervals. If the variation between any two readings exceeds 10%, the series will be repeated after an additional 5-min rest. The mean of three stable measurements will be recorded as the reference SBP.

### Intraoperative monitoring

Standard monitoring will be established upon operating room arrival. Prior to induction, advanced monitoring will be initiated with a radial arterial catheter under local anesthesia for continuous blood pressure monitoring and arterial blood gas sampling. The arterial line will be connected to a LiDCOrapid system (LiDCO Ltd., United Kingdom) for stroke volume (SV), stroke volume variation (SVV), cardiac index (CI) measurement to guide goal-directed fluid therapy (GDFT) and vasopressor titration. Depth of anesthesia will be monitored using the Bispectral Index (BIS). All arterial pressure (SBP, DBP, MAP) and heart rate (HR) will be recorded electronically, with values extracted every 5 min for subsequent analysis.

### Anesthesia induction and intervention

After preoxygenation, general anesthesia will be induced with intravenous sufentanil (0.2–0.3 μg/kg), etomidate (0.1–0.2 mg/kg), and rocuronium (0.6 mg/kg). An i-gel supraglottic airway will be inserted, and mechanical ventilation will be commenced.

Hemodynamic management will begin immediately post-induction. In the individualized management group, a continuous peripheral intravenous infusion of norepinephrine (10 μg/mL) will be started at 0.02 μg/kg/min and titrated to maintain SBP within ± 10% of the baseline, with a maximum dose of 0.2 μg/kg/min. In the standard management group, intermittent intravenous boluses of norepinephrine (10 μg) will be administered as rescue therapy only if hypotension occurs (defined as SBP < 90 mmHg or a > 30% decrease from baseline). For all patients, the MAP will be maintained at ≥ 65 mmHg as a universal minimum target [17]. If target blood pressure cannot be maintained at the maximum dose, rescue therapy (e.g., dopamine 2–5 μg/kg/min or epinephrine 0.01–0.05 μg/kg/min) will be initiated based on real-time hemodynamic monitoring. Finally, an ultrasound-guided supra-inguinal fascia iliaca compartment block (S-FICB) with 35 mL of 0.25% ropivacaine will be performed on the surgical side for postoperative analgesia.

### Anesthesia maintenance

Anesthesia will be maintained with sevoflurane titrated to a BIS value of 40–60. Mechanical ventilation will be set to a tidal volume of 8 ml/kg with respiratory rate adjusted to maintain end-tidal CO₂ between 35–45 mmHg. Supplemental bolus doses of rocuronium, sufentanil, and remifentanil may be administered as needed.

GDFT will be guided by the LiDCOrapid system. A fluid challenge of 250 mL lactated Ringer’s solution will be administered over 10 min; an increase in SV of ≤ 10% will be considered indicative of fluid non-responsiveness, establishing the optimal SV. SVV will be maintained at ≤ 13% throughout surgery (S2 Appendix).

### Recovery of anesthesia

At the end of surgery, anesthetic administration is terminated and the patient is transferred to the postanesthesia care unit (PACU), where the same intraoperative hemodynamic management strategy is maintained. The laryngeal mask airway is removed once the patient responds to commands and demonstrates adequate spontaneous ventilation. Pharmacologic reversal agents are administered if indicated for persistent respiratory depression. Patients will be observed for at least 30 min after extubation and must achieve a modified Aldrete score of ≥ 9 before discharge to the ward.

### Postoperative analgesia

Upon ward or ICU admission, a standardized intravenous patient-controlled analgesia (PCA) protocol is initiated. The PCA solution contains sufentanil 1 µg/mL (100 µg diluted in 100 mL normal saline) and is programmed with a basal infusion of 2 mL/h, a bolus dose of 2 mL, and a 15-min lockout interval. Nurses instruct patients to self-administer boluses for breakthrough pain (NRS > 3). Respiratory rate, sedation level, and pain intensity are monitored and documented at 4-h intervals to ensure safety.

## Outcome

### Primary outcome

The primary outcome is a composite of at least one organ system dysfunction (including respiratory, cardiovascular, renal, and nervous system) in patients within 7 days after surgery. The occurrence and severity of organ dysfunction should be assessed daily and evaluated during follow-up assessments (For specific definitions of organ system dysfunction, please refer to the S1 Appendix).

### Secondary outcomes

The secondary outcomes encompass the components of the primary outcome, other postoperative clinical events (including systemic inflammatory response, infection, and thromboembolic events), intraoperative variables (hemodynamic data, fluid balance, and serum lactate levels), postoperative recovery measures (pain score, durations of ICU and hospital stay), and all-cause mortality within 30 days after surgery. More details are provided in the S2 Appendix.

Hemodynamic variables, collected from anesthesia induction until PACU discharge, will consist of the total dosage of norepinephrine administered; the incidence of intraoperative hypotension (defined as SBP < 90 mmHg or a decrease > 30% from baseline, or MAP < 65 mmHg); the total dosage of urapidil administered; the incidence of intraoperative hypertension (defined as SBP > 160 mmHg and/or diastolic blood pressure > 90 mmHg) [15]; and the occurrence and management of severe bradycardia (defined as heart rate < 40 beats per minute).

### Follow-up procedures

To ensure a comprehensive evaluation of the primary outcome within 7 days after surgery, a structured follow-up and data collection procedure will be implemented (Table 1). Blood samples are planned to be collected from all patients on postoperative days 1, 2, and 7. For patients discharged earlier, detailed guidance and assistance will be provided to facilitate their return for clinical assessment and blood sampling on day 7. If an in-person return is not possible, a blinded assessor will conduct a structured telephone interview to collect information on clinical symptoms. Concurrently, laboratory test results that are routinely available from other healthcare institutions during this period will be collected. The primary composite outcome will be adjudicated based on all clinical and laboratory data obtained up to postoperative day 7.

### Adverse events

Adverse events (AEs) primarily include respiratory depression, bradycardia, nausea and vomiting, chills, anaphylactic shock, and cardiac arrest. Any unexpected AEs occurring during the trial will be promptly managed. In the event of a serious adverse event (SAE), regardless of its relationship to the intervention, the investigator will document it in detail in the source records, complete the case report form (CRF), and sign and date the entries contemporaneously.

### Data collection and management

All participants will be evaluated at baseline. All data will be collected using paper-based CRFs. Hard copies of the CRFs will be stored in locked cabinets with restricted access. Data from the CRFs will be entered into a secure electronic database. Access to the database will be limited to authorized study personnel who have signed a confidentiality agreement. All participant personal information will be de-identified and kept strictly confidential throughout the study. Anonymized datasets may be made available for secondary research purposes upon reasonable request to the corresponding author and with approval from the institutional review board.

### Sample size estimation

The sample size calculation was based on the primary composite outcome of postoperative organ dysfunction. A previous cohort study reported incidences of 51.7% *vs*. 38.1% for standard and individualized management, respectively [15]. However, the particularly high vulnerability of older hip fracture patients is demonstrated by elevated rates of intraoperative hypotension (approximately 60%) [18], postoperative AKI (7.6% to 11.3%) [18–20], and myocardial injury (14%) [18], we anticipate a higher baseline risk in our standard care group and a potentially larger treatment effect.

Therefore, to ensure the study is adequately powered to detect a conservative and clinically significant effect, we assumed a 55% incidence of the primary outcome in the standard management group and a 30% incidence in the individualized management group. Using a chi-square test in PASS 15 software with a two-sided α of 5% and 90% power, a minimum sample size of 82 patients per group was calculated. To account for an anticipated 10% dropout rate, a total of 180 patients (90 per group) will be enrolled. This calculation employs a conservative effect size estimate, anticipating a higher baseline risk of organ dysfunction in our frail older hip fracture population compared to the referenced study in major abdominal surgery [15], thereby ensuring adequate power to detect a clinically meaningful difference.

### Statistical analysis

All analyses will be performed using SPSS (version 25.0; IBM Corp., Armonk, NY, USA). A two-sided *P*-value < 0.05 will be considered statistically significant. The primary outcome will be compared between groups using the chi-square test. To account for potential baseline imbalances, the primary analysis will be supplemented with a multivariable logistic regression model adjusting for age (as a continuous variable) and ASA physical status classification. The normality of continuous data will be assessed using the Shapiro–Wilk test. Continuous variables will be summarized as means with standard deviations or medians with interquartile ranges and compared using the independent samples t-test or the Mann–Whitney U test, as appropriate. Categorical variables will be presented as numbers (percentages) and compared using the chi-square test or Fisher’s exact test.

For secondary analyses, the relationships between the intervention and the primary outcome will be further explored using multivariable logistic regression, adjusting for key baseline covariates. Repeated-measures data (e.g., SBP over time) will be analyzed using generalized estimating equations. Time-to-event data will be analyzed with the Kaplan–Meier method and the log-rank test. The ITT principle will be applied for all analyses. Any missing data will be handled using complete-case analysis.

## Discussion

This randomized controlled trial is designed to evaluate whether an individualized blood pressure management strategy (targeting SBP within ± 10% of baseline) can reduce postoperative organ dysfunction more effectively than a standard reactive management strategy in older patients undergoing hip fracture surgery under general anesthesia. The underlying mechanism is hypothesized to be through preemptive stabilization of hemodynamics, thereby minimizing exposure to IOH and maintaining personalized organ perfusion pressures tailored to individual autoregulatory thresholds.

The primary goals of anesthetic management in hip fracture patients are to mitigate postoperative complications and facilitate early recovery and functional rehabilitation [21]. IOH is a well-established, independent risk factor for a spectrum of adverse outcomes, including AKI, myocardial injury, and postoperative delirium [22, 23]. Notably, cardiovascular events and AKI are leading contributors to the high 30-day mortality observed after non-cardiac surgery, with up to one-third of deaths potentially attributable to perioperative myocardial injury [23]. Consequently, optimizing intraoperative hemodynamics is paramount for improving outcomes in this vulnerable geriatric population [24].

General anesthetic agents induce vasodilation and reduce systemic vascular resistance, compromising cardiovascular compensatory mechanisms [25]. This is particularly detrimental in older patients with diminished physiological reserve and impaired autoregulation. Evidence consistently demonstrates that blood pressure deviations exceeding 30% from baseline are associated with prolonged recovery and increased mortality [26, 27]. This underscores the limitations of a one-size-fits-all approach using fixed thresholds (e.g., MAP < 65 mmHg or SBP < 90 mmHg) and supports the rationale for individualized targets based on preoperative baseline values.

In the previous study, Futier et al. [15] demonstrated that individualized SBP management during major abdominal surgery reduced postoperative organ dysfunction. We hypothesize that an individualized perfusion pressure management strategy, guided by baseline blood pressure and initiated preemptively before hypotensive events, may improve tissue oxygenation and microcirculation in older hip fracture surgery patients, thus mitigating multi-system ischemic injury.

The choice of norepinephrine as the primary vasopressor is based on its favorable pharmacological profile for this population. Unlike phenylephrine, which is a pure α-agonist and can reduce heart rate and cardiac output, norepinephrine possesses both α- and β-adrenergic activity. Its β1-effects provide mild positive inotropy, supporting cardiac output, while its vasoconstrictive effects improve venous return. This profile facilitates a more stable elevation in blood pressure with less impact on heart rate compared to pure vasoconstrictors. Furthermore, compared to ephedrine, which exhibits tachyphylaxis and variable effects [28], a continuous low-dose infusion of norepinephrine offers more predictable and titratable control, making it ideally suited for preemptive stabilization [29]. While concerns exist regarding vasopressor use and renal perfusion [29], the protocol employs low doses aimed at preventing profound hypotension, which is a greater threat to renal autoregulation. Evidence from animal models suggests norepinephrine can effectively restore microcirculatory flow during hypotension without compromising gut oxygenation [30].

This study has several limitations. First, as a single-center trial, the generalizability of our findings may be limited. However, the single-center design enhances internal validity by standardizing the intervention and perioperative care. Second, in compliance with our institutional ethics committee guidelines, we excluded patients over 85 years of age. While this enhances safety for our initial investigation, it means our results may not be directly extrapolated to the very older population, who represent a significant proportion of hip fracture patients and may derive substantial benefit from such management strategies. Future research should specifically address this age group. Lastly, the composite primary outcome, while designed to capture the overall burden of organ dysfunction, may mask specific effects on individual organ systems. However, it provides a holistic measure of patient-centered morbidity.

Despite these limitations, this trial is poised to provide high-level evidence regarding the efficacy of individualized blood pressure management in reducing postoperative multi-organ dysfunction among geriatric hip fracture patients. Our findings are expected to contribute to the development of standardized, evidence-based clinical protocols aimed at optimizing perioperative outcomes and improving survival and functional recovery in this vulnerable population.

## Supplementary Information

Supplementary Material 1. S1 Appendix：Supplementary Definitions 1.

Supplementary Material 2. S2 Appendix：Supplementary Definitions 2.

## Data Availability

No datasets were generated or analyzed during the current study.

## Abbreviations

IOH: Intraoperative hypotension
AKI: Acute kidney injury
MAP: Mean arterial pressure
SBP: Systolic blood pressure
SPIRIT: Standard Protocol Items for Randomized Trials
ASA: American Society of Anesthesiologists
NYHA: New York Heart Association
LVEF: Left ventricular ejection fraction
COPD: Chronic obstructive pulmonary disease
FEV1: Forced expiratory volume in 1 s
eGFR: Estimated glomerular filtration rate
ITT: Intention-to-treat
CFS: Clinical Frailty Scale
SV: Stroke volume
SVV: Stroke volume variation
GDFT: Goal-directed fluid therapy
BIS: Bispectral Index
S-FICB: Supra-inguinal fascia iliaca compartment block
PACU: Postanesthesia care unit
PCA: Patient-controlled analgesia
NRS: Numerical rating scale
AEs: Adverse events
SAE: Serious adverse event
CRF: Case report form
