# Supplementary material for: Individualized blood pressure management and postoperative organ dysfunction in older hip fracture patients: a study protocol for a single-center, randomized, controlled trial
Source: BMC Geriatr. 2026 May 2;26:863. doi: 10.1186/s12877-026-07594-5 (PMC13281500; doi:10.1186/s12877-026-07594-5)
Supplement: Supplementary file 1 — Supplementary Material 1. S1 Appendix：Supplementary Definitions 1. [file 12877_2026_7594_MOESM1_ESM.docx]

**Supplementary Definitions 1**

**Criteria for postoperative acute kidney injury**

The KDIGO staging criteria include the following three items：

1. An increase in SCr of ≥ 26.5µmol/L within 48 hours.
2. It is known or determined that the SCr has increased by ≥ 1.5 times the baseline value within the past 7 days.
3. Urine output within 6 hours is less than 0.5mL/kg/hr.

Any one of the above conditions can be diagnosed as acute kidney injury.

**Criteria for postoperative pulmonary complications**

1. Criteria for postoperative hypoxemia: Hypoxemia is defined as a PaO_2_ < 60 mmHg or SpO_2_ < 90% on room air.
2. Criteria for non-invasive ventilation: Non-invasive ventilation is considered for patients of presence and persistence for more than 30 minutes of hypoxemia (as defined above) and at least one of the following symptoms:
   1. A respiratory rate is higher than 30/min.
   2. Clinical signs suggest intense respiratory muscle work and/or labored breathing, such as the use of accessory respiratory muscles, paradoxical motion of the abdomen, or intercostal retraction.
3. Criteria for acute respiratory distress syndrome (ARDS): ARDS is defined according to the new global definition[1].
4. Criteria for postoperative pneumonia: Chest radiograph shows the presence of new and/or progressive pulmonary infiltrates plus two or more of the following：Body temperature ≥38.5°C or <36°C；Leukocytosis ≥ 12,000 WBC/mm³ or leukopenia < 4,000 WBC/mm³.

**Criteria for postoperative cardiovascular complications**

1. Criteria for myocardial injury after non-cardiac surgery (MINS) [2]：Elevated troponin due to myocardial ischemia during or within 30 days after non-cardiac surgery. Elevated troponin is defined as troponin I or troponin T exceeding the upper limit of the reference value.
2. Criteria for myocardial infarction (MI)[3]: MI is defined as a rise and/or fall in cardiac troponin with at least one value above the 99th percentile upper reference limit and any one of the following: symptoms of myocardial ischemia, new ischemic changes on the electrocardiogram, imaging evidence of new loss of viable myocardium or the identification of thrombosis on coronary angiography.
3. Criteria for acute heart failure[4]: Acute heart failure is identified by appropriate clinical history and examination and consistent cardiac ultrasonography.

**Criteria for postoperative neurological complications**

1. Criteria for postoperative delirium. The confusion assessment method (CAM) is divided into four main aspects: a. Acute changes or fluctuations in the state of consciousness; b. Inattentiveness; c. Disordered thinking; d. Change in the level of consciousness. Postoperative delirium can be diagnosed if one has both a and b, as well as either c or d.
2. The presence of postoperative altered consciousness by day 7 after surgery is determined clinically by the treating physician, and defined as a Glasgow Coma Scale (GCS) score of 14 or less (SOFA sub-score of 1 point or more in the neurologic component).
3. Acute ischemic stroke is defined as an acute new focal neurologic deficit with confirmation by CT scan and/or MRI.

**References**

1. Matthay, M.A., et al., *A New Global Definition of Acute Respiratory Distress Syndrome.* Am J Respir Crit Care Med, 2024. **209**(1): p. 37-47.

2. Devereaux, P.J. and W. Szczeklik, *Myocardial injury after non-cardiac surgery: diagnosis and management.* Eur Heart J, 2020. **41**(32): p. 3083-3091.

3. Lindahl, B. and N.L. Mills, *A new clinical classification of acute myocardial infarction.* Nat Med, 2023. **29**(9): p. 2200-2205.

4. McDonagh, T.A., et al., *2021 ESC Guidelines for the diagnosis and treatment of acute and chronic heart failure.* Eur Heart J, 2021. **42**(36): p. 3599-3726.
