# Supplementary material for: Individualized blood pressure management and postoperative organ dysfunction in older hip fracture patients: a study protocol for a single-center, randomized, controlled trial
Source: BMC Geriatr. 2026 May 2;26:863. doi: 10.1186/s12877-026-07594-5 (PMC13281500; doi:10.1186/s12877-026-07594-5)
Supplement: Supplementary file 2 — Supplementary Material 2. S2 Appendix：Supplementary Definitions 2. [file 12877_2026_7594_MOESM2_ESM.docx]

**Supplementary Definitions 2**

**Secondary outcomes**

- Postoperative complications include inflammatory response (systemic inflammatory response syndrome [SIRS]), infection (surgical site infection, sepsis), respiration (hypoxemia, acute respiratory distress syndrome [ARDS], need for non-invasive or invasive mechanical ventilation, pneumonia), nervous system (stroke and postoperative changes in consciousness), thromboembolism (deep vein thrombosis, pulmonary embolism), cardiovascular system (arrhythmia, myocardial injury, myocardial infarction, acute heart failure)，and renal complication (acute kidney injury, acute renal failure).

1. **Criteria for SIRS [1, 2]**

SIRS is defined by two or more of the following:

1. Core temperature >38°C or <36°C. (Core temperature was rectal or tympanic). If oral, inguinal or axillary temperatures were used, 0.5°C were added to the measured value.
2. Heart rate >90/min. If patient had an atrial arrhythmia, record the ventricular rate. If patients have a known medical condition or are receiving treatment that would prevent tachycardia (for example, heart block or beta blockers), they must meet two of the remaining three SIRS criteria.
3. Respiratory rate > 20/min or a PaCO_2_ <32 mmHg (4.3 kPa) or mechanical ventilation for an acute process.
4. White Blood Cell (WBC) count of >12 ×10^9^ /L or <4 x 10^9^ /L.
5. **Criteria for surgical site infection (SSI) [3]**

It is mainly divided into superficial incisional SSI, deep incisional SSI and organ/space SSI. It is defined according to the criteria of the CDC/NHSN 2023 standard.

1. **Criteria for deep vein thrombosis and pulmonary embolism**

The deep vein thrombosis and pulmonary embolism after surgery is defined according to the criteria of deep vein thrombosis and pulmonary embolism[4].

- Total intraoperative blood loss

The total intraoperative blood loss of the patient is calculated using the hematocrit (Hct) method. The Hct baseline value is recorded based on the preoperative blood gas analysis (Hct_1_), and the Hct value is detected again by blood gas analysis after the operation (Hct_2_). Total blood loss = (Hct_1_ – Hct_2_) × weight ×70÷ Hct_1_).

- The total intraoperative fluid volume

The total infusion volume of the patient from the start of anesthesia induction to the end of the surgery, including the volume of crystals, colloids and blood transfusions, as well as the total output volume, including blood loss and urine volume.

- Postoperative pain score

Recording the NRS score of the patient's pain after regaining consciousness in the resuscitation room and 1 and 2 days after the operation.

- Duration of ICU stay

The total duration of a patient's stay in the ICU from admission to discharge.

- Hospital stay

The duration of hospital stay for the patient from admission to discharge during this treatment.

- All-cause mortality within 30 days after surgery

The death of patients caused by any reason from the day of surgery to 30 days after surgery. The sources and confirmation methods of death information may include: medical records, death certificates, and telephone follow-ups.

**Trial settings for intraoperative fluid administration** [5, 6]

All patients use LiDCOrapid system (LiDCO Ltd., United Kingdom) for hemodynamic monitoring to guide goal-directed fluid therapy regimens, and lactate Ringer's solution is intravenously infused with infusion pumps at a rate of 4 mL/kg/h to maintain fluid requirements. Before anesthesia induction, all patients undergo a fluid challenge, that is, within 10 minutes, 250ml of pre-heated lactate Ringer's solution is intravenously infused. If stroke volume (SV) increases by less than 10%, SV is considered the optimal SV at this time, and the stability of SV should be maintained during the operation. If SV increases by ＞10% for more than 5 minutes, continue to infuse 250 ml of lactate Ringer's solution, with a total volume not exceeding 500ml (eFig.1).

After anesthesia induction, the stroke volume variation (SVV) is used as the assessment index. The SVV is maintained ≤13%. When SVV ≤13%, input lactate Ringer's solution at the background volume. If SVV ＞13% for more than 2 minutes, an additional 250ml of lactate Ringer's solution is infused (eFig.2).

eFig.1.


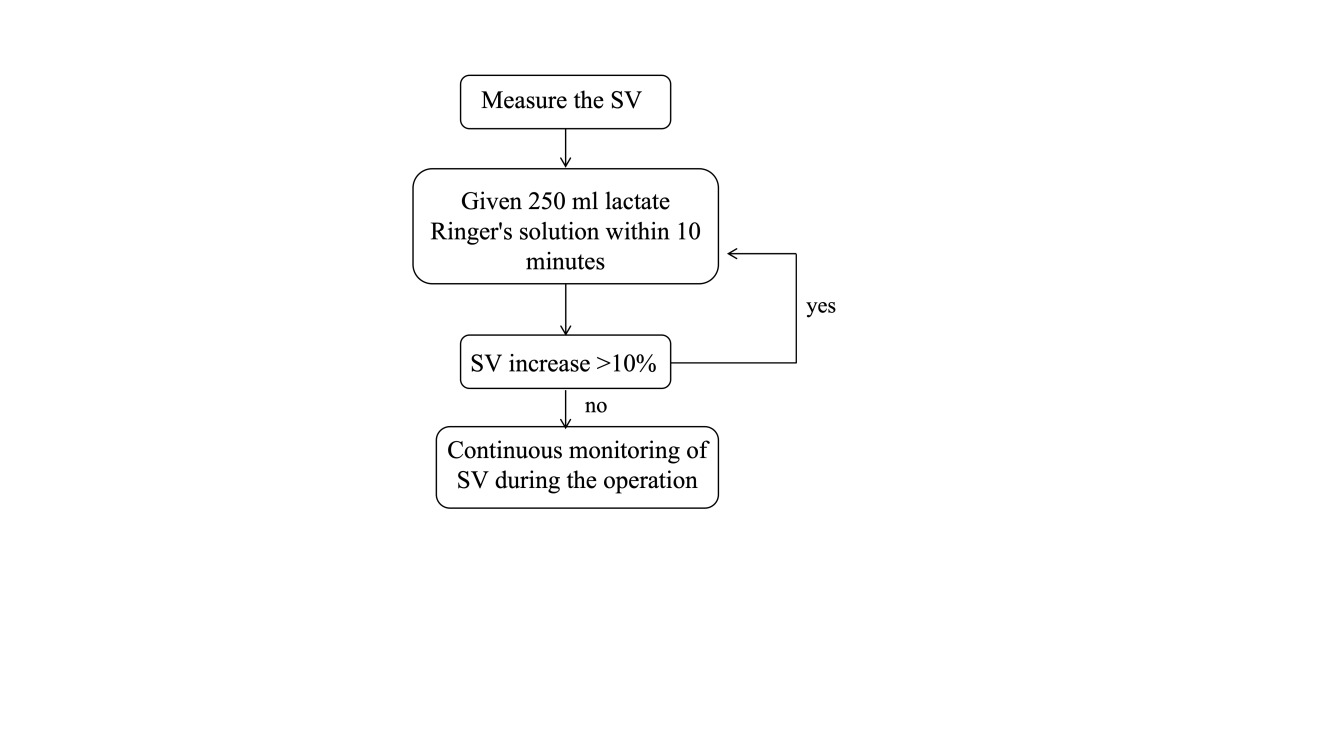


eFig.2


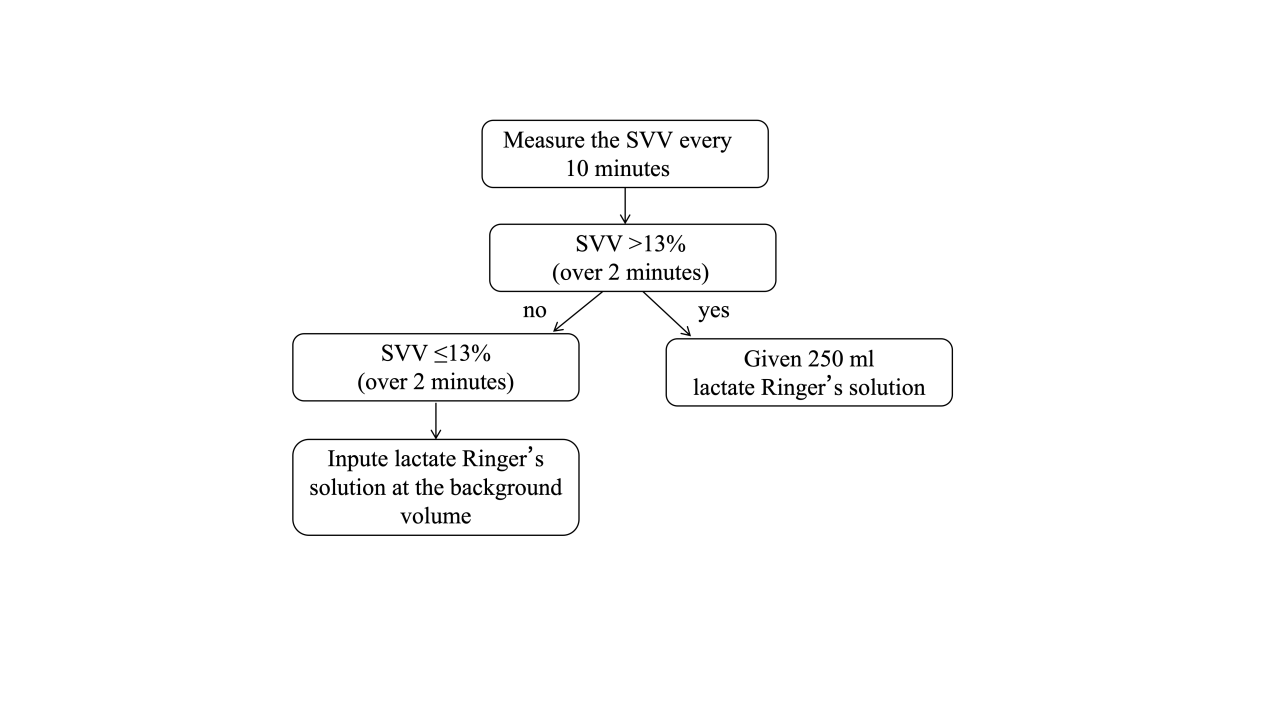


**References**

1. Schlapbach, L.J., et al., *International Consensus Criteria for Pediatric Sepsis and Septic Shock.* Jama, 2024. **331**(8): p. 665-674.

2. Mai, H., et al., *Identification of a Susceptible and High-Risk Population for Postoperative Systemic Inflammatory Response Syndrome in Older Adults: Machine Learning-Based Predictive Model.* J Med Internet Res, 2024. **26**: p. e57486.

3. *Surgical Site Infection Event (SSI)*. 2025; Available from: <https://www.cdc.gov/nhsn/pdfs/pscmanual/9pscssicurrent.pdf>.

4. Di Nisio, M., N. van Es, and H.R. Büller, *Deep vein thrombosis and pulmonary embolism.* Lancet, 2016. **388**(10063): p. 3060-3073.

5. Liu, F., et al., *Randomized controlled trial of regional tissue oxygenation following goal-directed fluid therapy during laparoscopic colorectal surgery.* Int J Clin Exp Pathol, 2019. **12**(12): p. 4390-4399.

6. Moppett, I.K., et al., *LiDCO-based fluid management in patients undergoing hip fracture surgery under spinal anaesthesia: a randomized trial and systematic review.* Br J Anaesth, 2015. **114**(3): p. 444-59.
